# Phylomemetic Patterns in Science Evolution—The Rise and Fall of Scientific Fields

**DOI:** 10.1371/journal.pone.0054847

**Published:** 2013-02-11

**Authors:** David Chavalarias, Jean-Philippe Cointet

**Affiliations:** 1 Complex Systems Institute of Paris Ile-de-France (ISC-PIF), Paris, France; 2 CAMS, CNRS - EHESS, Paris, France; 3 INRA-SenS, INRA, Marne-la-Vallée, France; Max Planck Institute for the Physics of Complex Systems, Germany

## Abstract

We introduce an automated method for the bottom-up reconstruction of the cognitive evolution of science, based on big-data issued from digital libraries, and modeled as lineage relationships between scientific fields. We refer to these dynamic structures as *phylomemetic networks* or *phylomemies*, by analogy with biological evolution; and we show that they exhibit strong regularities, with clearly identifiable phylomemetic patterns. Some structural properties of the scientific fields - in particular their *density* -, which are defined independently of the phylomemy reconstruction, are clearly correlated with their status and their fate in the phylomemy (like their age or their short term survival). Within the framework of a *quantitative epistemology*, this approach raises the question of predictibility for science evolution, and sketches a prototypical life cycle of the scientific fields: an increase of their cohesion after their emergence, the renewal of their conceptual background through branching or merging events, before decaying when their density is getting too low.

## Introduction

How is science evolving? Is it possible to map the landscapes of science and its transformations? Can we automatically decipher the history of a research field, monitor emerging fields and detect research hybridization events? Numerous theories, and more or less conceptual models of science evolution have been contemplated in the philosophy of science [1]–[3], and in Science & Technology Studies [4]–[6]. One of the most recent theories underlines the ever-changing nature of science and the research process [7]. According to Nowotny *et al.*, science has recently entered a new *mode*, in which knowledge is generated within a wider context of applications, bringing trans-disciplinarity, defined as the circulation of tools, theoretical perspectives and people to the forefront. However, whatever the driving forces behind science evolution, there is still a lack of empirical instruments allowing researchers to fully understand the nature of these transformations.

Meanwhile, the recent ICT revolution has, at an ever-growing pace, opened up new digital spaces, offering new opportunities to track the dynamics of knowledge through the examination of its digital trails. Scientific research pioneered this trend towards a *stigmergic society* when the “Science citation Index” was first published in 1961 [8]. The current massive availability of online data related to science production provides a unique opportunity to *map the ever-fluctuating scientific landscape* and to unveil regular dynamical patterns from large-scale longitudinal observations. Nevertheless, we still need specific data-mining methods to extract patterns from these large databases.

In this article, we propose a method that reconstructs the dynamics of scientific fields, and we apply it to two large-scale case studies: “embryology research” and “networks in biology” corpora. We define scientific fields as sets of “keywords” delineating a research area, in the same way that journals or conferences describe the scope of their publications or presentations. These fields evolve over time: new concepts are introduced, and new directions of research are initiated, resulting in the emergence of new fields, some of which may split or merge with other fields, or may simply disappear if the underlying scientific community loses its thematic coherence.

This lively evolution of science, featuring innovations, cross-fertilization and selection, is suggestive of an analogy with the evolution of living organisms. We propose an adaptation of the concept of the phylogenetic tree, and combine it with the Richard Dawkins intuition of *meme* [9], to refer to *phylomemetic networks* (or *phylomemy*), which describes the complex dynamic structure of transformation of relations between terms. The concept of “phylomemetic network” is used by analogy to biological phylogenetic trees, which account for evolutionary relationships between genes. We do not make any assumption concerning the type of dynamics underlying the evolution and diffusion of terms. As such, contrarily to previous works in line with the memetics theory [9], which have already coined the term (*e.g.* [10]), we do not claim that cultural entities (memes) evolve following the same laws of selection as biological replicators (genes) do. A discussion about the ontological nature of terms, their transformations and the dynamics of their relationships would require *per se* a proper paper.

These structures allow us to focus on the dynamics of science, whereas most empirical studies usually focus on the structure of science and think of datasets as crystallized snapshots of science production at a given period. These science phylomemies describe how scientific fields evolve, and provide a convenient model to answer questions such as: what are the most striking regularities in science evolution? What are the emerging fields at a given time? From which scientific fields does a new field inherit its intellectual background? Which “paradigmatic” shifts have been the most dramatic? etc.

In this article, we show that, from this perspective, co-word analysis is a suitable approach. We firstly describe the general methodology which has been devised to enable a bottom-up reconstruction of science phylomemy, and then demonstrate that this approach provides evidence of strong regularities in science evolution.

## Materials and Methods

**Figure 1:**
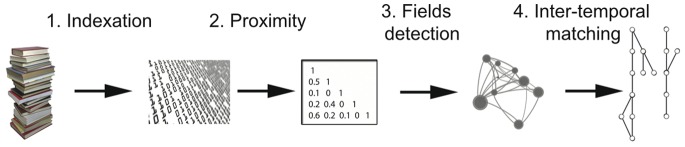
Steps contributing towards the reconstruction of a phylomemy.

The reconstruction of science phylomemy methodology can be divided into four steps (*cf.* Fig. 1):

1. Mining and indexation of key-phrases inside a corpus,
2. Measuring proximities between key-phrases,
3. Clustering key-phrases into scientific fields,
4. Inter-temporal matching between thematic fields.

In the following, we briefly describe steps 1 to 3, as well as the precise manner in which they were implemented for our case studies. These first steps are classical in science mapping methods. Conversely, the 4*^th^* step is specific to science dynamics mapping and is described exhaustively. Our goal is not to show that all of the steps in this workflow are optimal for the tracking of science evolution, but rather to demonstrate that this bottom-up approach is well-adapted to revealing the robust patterns of science evolution.

### Key-phrase Extraction

In order to propose methods which can be scaled to various data sources, we chose to work with aggregated co-occurrences data relevant to terms in documents, which can easily be extracted from digital corpora using classical indexing technique. Our main dataset is based on a scientific publications corpus related to research in *embryology*. It comprises approximately 200,000 articles, extracted from the “Thomson Web of Science” (WoS), in which the stem “embryo” is used in their title or abstract.

Co-word analysis critically depends on the initial set of terms chosen for the study and can be biased by the “indexer effect” [11]–[13]: library managers may choose keywords which are too general, place incorrect emphasis on some terms, etc. For this reason, we performed an automatic lexical extraction of the set of terms to be considered based on titles and abstracts in our datasets.

The Natural Language Processing (NLP) tools we apply for the purposes of lexical extraction allows not only simple terms, but also multi-terms (up to 4, also called n-grams) to be identified. Although the automatic extraction of multi-terms is a classical task in NLP, the existing tools are not always well suited when one wishes to extract only domain-specific noun phrases, that is groups of relevant terms featuring both high unithood and high termhood, as defined in [14]. For that reason, we designed our own workflow for processing of the textual data, which is described in Supporting Information S1. An improved version of this workflow is available online in the open access tool CorText Manager (http://manager.cortext.net).

This linguistic processing chain produced a list 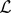 of 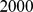 n-grams which should constitute the most salient terms in embryology science. This list was checked manually by experts in the field, who identified a little more than ten considered to be irrelevant. In view of this this high rate of relevance, we chose to keep the full list, in order to minimize the influence of human intervention in the process. These terms were then indexed in the 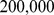 retrieved titles and abstracts dating from 1990 to 2010, to build up the set of co-occurrence matrix 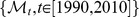 based on this terms list. The co-occurrence 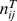 between two terms 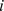 and 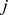 over a period 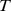 is simply defined as the number of articles published during this period which mentioned both of the terms 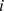 and 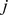 at least once in their abstract or title. In the case of our dataset, 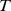 is taken to be one year, which is the finest resolution which can be achieved for this parameter.

In order to test the robustness of our results with respect to dataset variations and the quality of the terms list, we performed a similar analysis on a second dataset, but choose to remove irrelevant terms. This second dataset, called “bio-networks”, is more trans-disciplinary. It comprises a biomedical corpus related to the research which recourses to the concept of networks. It includes approximately 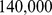 articles extracted from Medline (http://www.ncbi.nlm.nih.gov) using the stem “netw” in their title or abstract. A list of 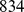 salient terms have been extracted from this corpus. As presented in the Supporting Information S2, all our results were reproduced on this second dataset.

All terms lists and co-occurrence data used in this paper are available online at http://hdl.handle.net/1902.1/19062.

### Measuring Proximities

The use of co-occurrence data in scientometrics assumes that the greater the probability of two elements co-occuring in the same article, the more strongly they are related. This co-occurrence data can be of various types and may connect authors - co-authorship networks [15], references - co-citation networks [16], or terms - co-word networks [17], [18]. We focus on the latter type of data, in the framework our of co-word analysis. In this approach, co-occurrences of terms are computed over large corpora. A graph is then generated, on which the nodes represent the terms, and the edge strength represents their alleged similarity, which is a function of the co-occurrence data. Higher level structures reflecting various domains of science are then derived using clustering methods, by analyzing patterns within this graph.

Several proximity measures based on co-occurrence data have been introduced in the science mapping literature. Their function is to capture specific type of preferential relationship between two terms - see [13] for a comprehensive review. Some co-occurrence measurements are direct (mutual information, inclusion index, proximity index), whereas others are indirect (cosine). Direct measurements take only the number of raw co-occurrence between two nodes into account, whereas indirect measurements account for the global distribution of co-occurrences of the two nodes over all nodes. In the present study, we considered the conditional probability measure 
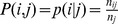
 which has an intuitive and straightforward interpretation: the proximity between 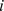 and 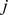 measures the probability of observing term 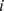, making use of the prior knowledge that other articles also use the term 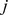. We also tested other measurements, which generally led to qualitatively similar results. However, it is important to note here that there exists at least one proximity measurement which reveals the robust patterns of science evolution.

### Clustering

Starting from a set of terms 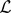 to be mapped, the conditional probability transforms the co-occurrence matrix into a non symmetric proximity matrix 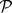.

We then transformed this proximity matrix into an adjacency matrix characterized by a value equal to 1 at all locations where the proximity between two terms greater than a threshold of 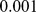, and equal to 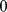 elsewhere. Although the use of a proximity threshold of 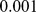 is arbitrary, it eliminates almost all irrelevant relations relationships. The adjacency matrix is then transformed into a network 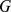.

This new graph 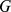 can be further analyzed using clustering methods, in which the clusters are designed to represent scientific fields described as sets of strongly related terms that contextualize each other’s meaning, with some being more specific, and others more generic. Several clustering methods have been proposed in the literature and extensively tested for science mapping, *e.g.* k-means clustering [19], [20], Self-Organized Maps [21], information flow [22]. When applied to 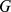, they may correspond to slightly different definitions of the structural features that sets of terms should meet to represent a given scientific field.

We chose a very simple and comprehensible methods to define our clusters: in line with [23] we define scientific fields as *directed cliques* of terms in 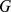. One advantage of this approach, beside its clarity, is that it allows for the overlapping of clusters, and a given term can thus be represented with various meanings in different scientific fields. Nevertheless, one of the drawbacks of the clique detection approach is that is does not take each link’s weighting into account: weak and strong links are treated equally. For this reason we chose to build a non-weighted network. We used the CFinder algorithm [24] to perform the clique detection, available at http://www.cfinder.org. In the following, the set of directed cliques (or *scientific fields*) detected over a period 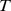 will be noted 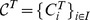. To ease the interpretation of the results, we consider periods of one year. It is however possible to consider longer periods, in order to gain in statistical significance by grouping a larger number of documents per period. As an example, we investigated a case using periods of three years, with qualitatively the same results.

### Tracking Meso-dynamics

One of the most direct techniques for the study of science evolution is to track how new associations between terms change the composition of scientific fields.

Term level dynamics are too noisy and polysemic to provide a reliable and synthetic account of the evolution of scientific activity. Therefore one should primarily analyze the “meso-level” of fields: how are different fields cross-fertilizing, is a given field of activity growing or declining, can we predict when a field is going to burst or collapse? Once this structural analysis has been achieved we can focus on, and analyze the structure at the micro-level; examining for example how terms circulate through fields. Thus, in order to characterize how the “structure” of science evolves, we focus on its *meso-dynamics*, which is equivalent to describing the transformations which occur between clusters, at successive periods of time.

The answer to this problem is far from straightforward. A scientific field, represented by a cluster 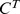 at a given period of time 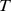, can undergo several kinds of compositional transformation, requiring different representations in the following periods: it can gain new terms, or loose others, merge with an other field, split or die. Two successive periods can feature very different sets of scientific fields. However, even two different scientific fields observed at two successive periods may potentially overlap and share a common scientific background. A scientific field can have several “offsprings” in the next period and its conceptual legacy may be found in several domains of investigation from the previous period.

The reconstruction of these inheritance patterns will provide a global overview of the dynamics caracterizing the transformations of large scientific domains. However, such patterns cannot be thought of as simple lineage trees, and we expect hybridization events between fields of research to occur whenever, in particular, two fields merge into one single field. Thus, the discrete transformation events at stake in the evolution of science fields can be represented as a network connecting successive fields. This is hereafter referred to as a *phylomemetic network* (or *phylomemy*).

**Figure 2:**
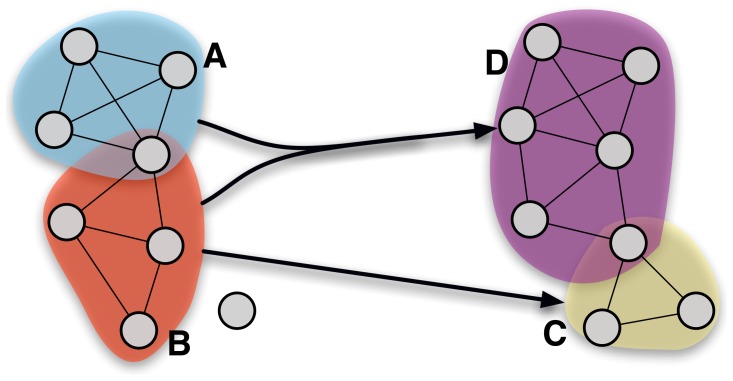
Inter-temporal fields matching.

To achieve suitable inter-temporal matching between fields, for each field 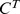 built over the period 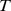 we need to find the field or union of fields from which it has inherited (*cf.* fig. 2). We assume that the time scale of the transformation of scientific fields is sufficiently slow to allow our empirical observation device to track “infinitesimal” transformations - meaning that the characteristic time scale over which we observe those transformations (typically 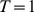 year) is greater than the fields’ actual pace of transformation (note that this principle of continuity had already been proposed a long time ago by Simmel [25] to track the “persistence” of social groups). We are thus trying to identify the past field or the combination of past fields at period 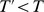 (since we allow for merging events) that would explain the 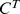 compositions in the most economic way - that is to say, the link between 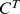 and its parent(s) involving the smallest number of changes (terms which are added or removed).

Formally, we can define an operator 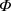 which for every period 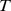 and every 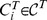 defines predecessors in the phylomemetic network as a subset 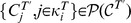 of fields present during the previous period 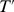, such that:
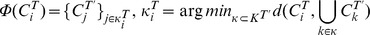



Since it would seem incorrect to match two fields having very few terms in common, even though no better matching can be found, we need to define a threshold above which the match is considered to be satisfactory (*i.e.* 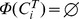 when 
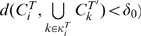
). We shall call this threshold 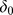. The threshold can be tuned, by systematically applying a minimum of similarity and a threshold sensitivity analysis, to guarantee the robustness of the results.

The most simple and also the most classical measurement that can be used to compare two sets of terms is the Jaccard similarity on field terms, which is the inverse of the “transformation index” introduced for similar purposes by Callon in [26], [27] also proposed a reconstruction method for social group (co-authorship and phone call network) evolution based on the same inter-temporal distance. Their general method is nevertheless different since it allows only linear lineages to be built, with the ascending and descending degrees of a cluster always having a value less than 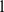.

Given two fields 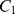 and 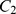 defined as a set of terms (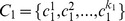, 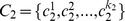), the Jaccard similarity is defined by: 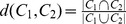.

Fig. 2 illustrates the matching procedure. It represents two successive sub-networks with the same set of nodes between two time steps. The two successive periods present distinct cluster sets : 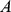 and 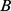 at time 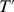 and 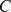 and 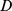 at time 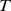. Note that one node belongs to two different clusters at time 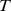. The aim is to determine from which fields or union of fields 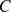 and 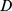 may be descending. It is straightforward to check that field 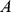 is the closest to cluster 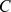 (*i.e.* 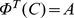). Even if two nodes were removed from 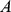 while one node was added, the similarity between 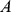 and 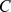 (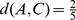) is still the best possible and offers the best matching. The case of 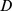 is more delicatesince there are three possible scenarios: 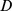 may inherit from 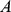, 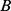 or 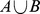. By computing the distance corresponding to each scenario, we get: 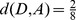, 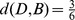 and finally 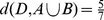. As it is most likely that 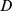 inherits from the merging of the two preceding fields 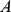 and *B*, we conclude that 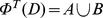.

### Phylomemetic Branches

**Figure 3:**
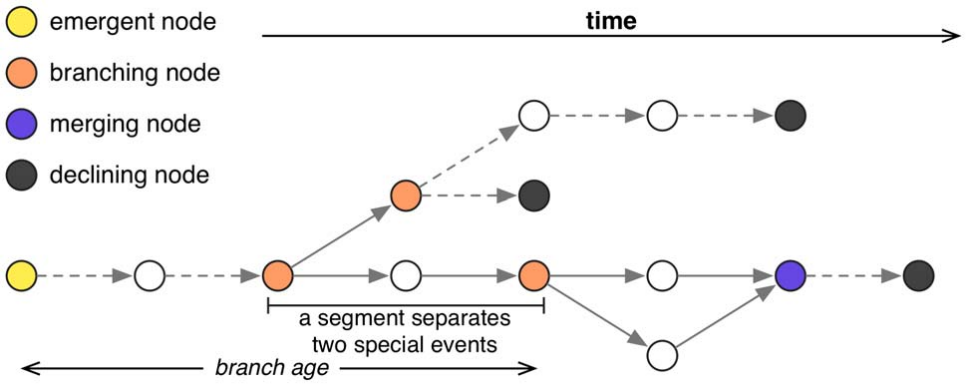
Example of a branch featuring branching and merging nodes.

**Figure 4:**
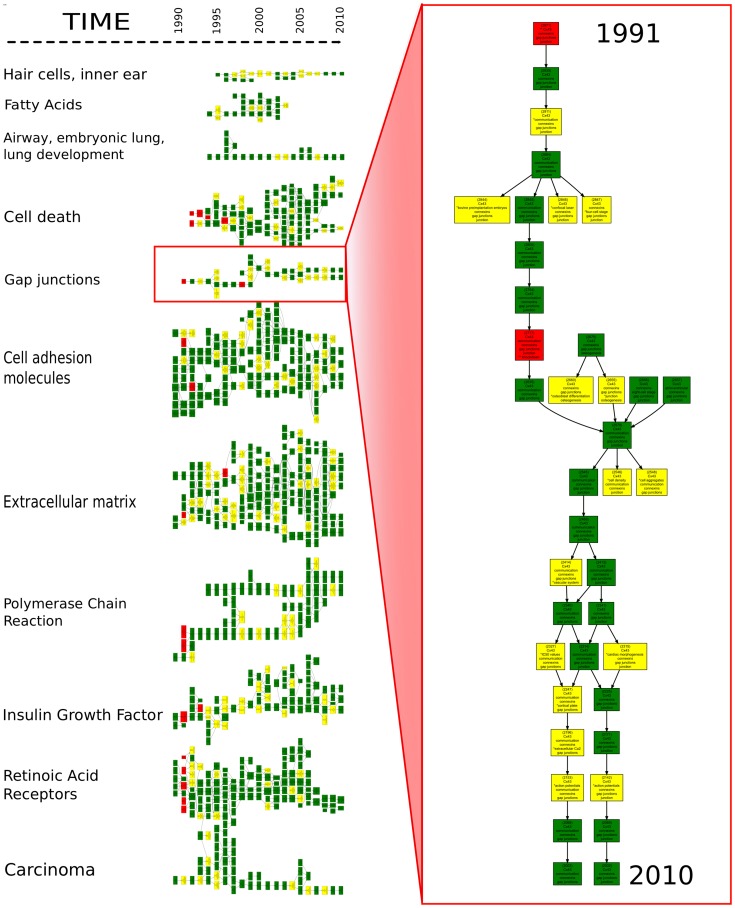
Sample of the phylomemy reconstruction for 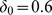. The phylomemetic branches naturally cluster the scientific fields into large areas of research. The branches presented in this figure have been labeled by their most commonly occurring terms (gap junction, extra cellular matrix, etc.). Time flows from left to right (from 1991 to 2010). Color coding has been used to highlight the existence of emerging terms (in red) or recombinations (in yellow) in clusters (*cf.* the Results section): a term associated with two **stars indicates that it is emerging, whereas one *star indicates that it is a recombination.

Before describing the structure of phylomemetic networks, various definitions need to be given, as summarized in Fig. 3. Connected components of the phylomemetic network are called *branches*, and generally correspond to large, clearly-cut domains, that is a set of scientific fields that have evolved with a common scientific background, but which can potentially address many different issues. For each branch, the most frequent terms are considered to automatically generate a label and acquire a general description of the issues studied in these large domains (some example are given in Fig. 4).

A *sub-branch* from field 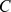 will refer to the sub-lattice formed by all the descendants of 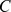. The distance between a given field and its most distant antecedent field defines its *age*. Nodes in the phylomemetic network featuring at least two sons are called *branching nodes*. Conversely, *merging nodes* have at least two fathers. These two types of node will be categorized as *special events*. *Segments* are linear sub-branches separating two *special events* (*cf.* Fig. 3). Qualitatively, a relatively cohesive thematic orientation is conserved in segments, whereas *special events* correspond to more dramatic changes such as branching, hybridization, or both.

Obviously, the structure of the phylomemetic network depends on the threshold 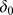. For low values of 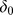, a small number of large branches collects most of the fields in the phylomemy. Since the nodes have a high degree, the coverage of the phylomemetic network (proportion of fields in the phylomemy having at least one parent or one child) is high. However, for low values of 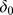, a significant proportion of the links in the phylomemy have a low weighting, and the inter-temporal matching is of low significance.

For higher values of 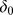, the inter-temporal matching is of high significance and a large number of branches is observed, although the lack of connectedness of the phylomemetic network tends to make them smaller. Consequently, the field coverage is poor. There is thus a set of intermediate values of 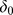 for which the branches provide good subdivision of the studied corpora into large, consistent research domains, while at the same time leading to satisfactory field coverage and relevant inter-temporal matching.

In order to discover regularities in the evolution of science, we need to find patterns which are relatively stable, for a large range of 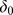 values within this intermediate domain. In the following, our results are presented for 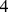 different values of this threshold, ranging from 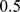 to 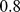. In order to verify that the discovered patterns are not specific to a particular corpus or to a particular choice of the domain under study, the same analyses were also applied to a second dataset and to an alternative text-mining technique, with similar results (*cf.* Supporting Information S2).

### Related Work

To our knowledge, four other papers address the modeling of scientific field transformations based on bibliometric data [28]–[31]. [28], [29] study journal citation data, which provide a different level of analysis, yet complementary to co-occurrence analysis, and leads to a lesser resolution than term co-occurrence analysis for the reconstruction of science evolution. There are also significant differences between [30], [31] and our approach regarding the initial methodological step. [31] directly mines raw textual content but does not involve advanced semantic pre-processing: every monograms is taken into account as primary material. This strategy may imperfectly reflect the richness of scientific landscapes. As for [30], publications are described through *PACS numbers* which offer a general top-down scheme for categorizing Physics literature. On the contrary, we propose an entirely bottom-up mining and indexation strategy. Hence our method is truly generic, as it can be applied to any domain of science and irrespective of the available metadata, paving the way to inter-domain comparison of dynamical patterns of science evolution. Moreiver, this genericity also makes it possible to deal with other kinds of media, like the blogosphere or the newspapers [32]. But more importantly, [29], [31] essentially focus on reconstruction methodology or visualization issues, ignoring the analysis of regularities of the reconstructed dynamics.

On the contrary, [30] do focus on the analysis of dynamical patterns of science evolution. However, the reconstruction strategy by Palla et al. [27], does not actually allow to account for forking and merging events. They consider strictly linear lineages (one son and one father at most for each cluster), which contrasts with the rich lattice structure resulting from our methodology. The dynamical structure obtained by [30] is thus simpler and does not allow to elucidate fine-grained patterns; for example around special events such as merging and branching. Moreover, Herrera et al. essentially analyze the evolution of the size of scientific fields (measured as the different number of PACS codes gathered within a cluster), and the activity, which is deeply correlated to size. Although fields size does exhibit clear patterns at an aggregated level, it seems less informative in our case of study to understand finer-grained dynamics, as demonstrated in Supporting Information S3. For example, we observed that field size grows with branch age, but is a poor predictor for the declining rate.

## Results

We performed a phylomemy reconstruction on the WoS database focusing on studies of embryology between 1991 and 2010 (see Fig. 4 for an example and the appendix for the delineation of the corpus and terms list). After the removal of interlocked clusters, the phylomemetic network of all fields of more than four elements features 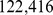 non-isolated nodes. It comprises different disconnected components, corresponding to large domains of embryology clustered in a time-consistent manner.

### Phylomemetic Patterns and Cluster Density Index

At first glance, Fig. 4 reveals interesting qualitative patterns. A strong disparity can be noticed between branches, with respect to the proportion of special events, with some branches being more linearly arranged than others. However, there is also temporal heterogeneity within the branches, in terms of the distribution of special events, which suggests that most domains of science undergo successive cycles of transformation during their evolution.

In the following, we search for the presence of dynamic patterns characterizing the evolution of science, and try to understand how these dynamic patterns are correlated, independently of the phylomemetic network, with the structure of the fields as they are measured at each time step. The objective is to gain some insight into questions such as: Are there early-warning signals prior to special events? Can we predict the probable fate of a scientific field? Or at a more microscopic level: what is the role of conceptual innovations in science evolution dynamics?

We focus on describing critical transition occurrences (merging, branching, etc.) in phylomemetic networks, according to the statically measured properties of the fields that undergo such transformations. For that purpose, we compute the *density index* of each field [26]: *“[Density] characterizes the strength of the links that tie the words making up the cluster together. The stronger these links are, the more the research problems corresponding to the cluster constitute a coherent and integrated whole.”* The density index for a cluster 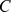 is defined by: 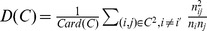. It is interesting to note that Michel Callon had already speculated that were was a relationship between a cluster’s density and its possible fate: *“It could be said that density provides a good representation of the cluster’s capacity to maintain itself and to develop over the course of time in the field under consideration.”* Although at the time this index was introduced, it was impossible to investigate science evolution on a large scale, the possibility of making such computations is now within reach.

We computed the density index of all the clusters obtained with the above reconstruction method. This density index was then normalized on a yearly basis, to allow inter-temporal comparisons to be made and to avoid side effects resulting from to variations in scientific community size, publication rate or corpora coverage. In the following discussion, the term *density* refers to this normalized density, that is to say: the raw value of field density divided by the mean density of all the fields detected during the same period of time. Callon et al.’s intuition suggested that high-density clusters could have a greater probability of being maintained over time than low density clusters. This intuition is empirically confirmed and even strengthened by our study.

**Figure 5:**
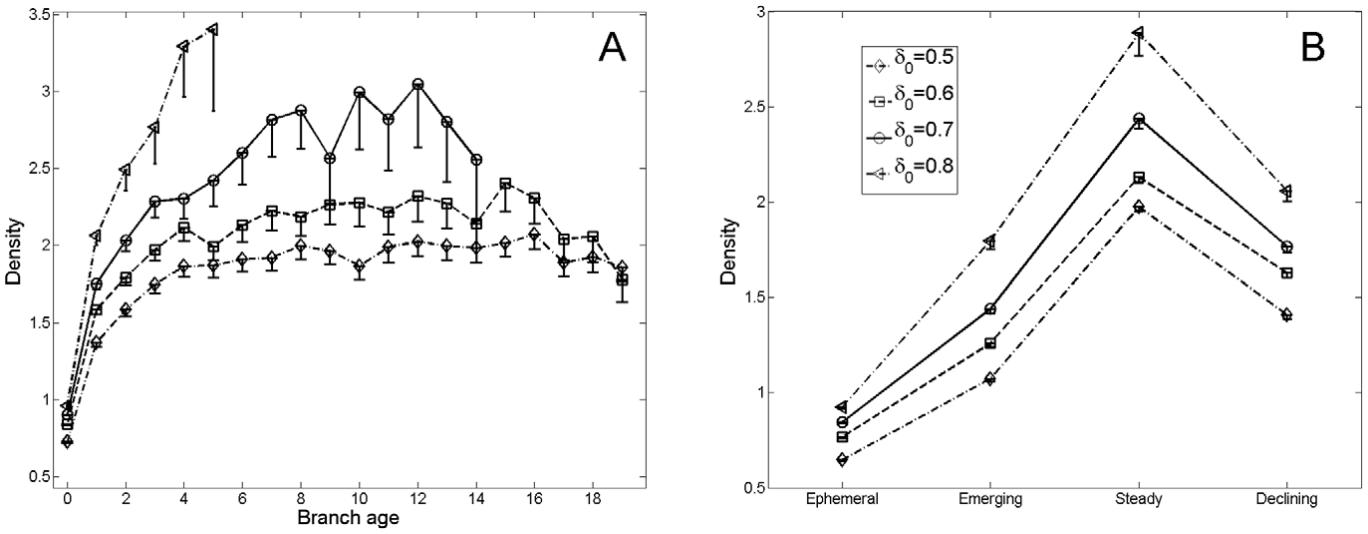
Relation between fields density and their age. **A.** Variation of the mean density depending on the branch age, for different values of threshold 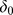. **B.** Dependence of the mean density on the fields’ position in the phylomemy. Fields in the phylomemy have a much higher density than ephemeral fields, and their density distribution suggests trends in the “life cycle” of thematic fields: the density grows when a new field is emerging, and decreases when the field starts to be neglected by the community. Error bars represent the 95% confidence interval. Only lower bars are plotted for better visibility.

We first plotted the average density of the fields against their branch age (see Fig. 5-a). For a large range of values of 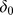, the density is positively correlated with the age of the current field: long lasting fields tend to feature a density far higher than the average value.

To further test this intuition, we introduced a different way of categorizing fields, according to their position in the phylomemetic network. Fields can be: *ephemeral* (no father nor child), *emerging* (no father, at least one child), *steady* (at least one antecedent and some descendent(s)) and *declining* (some antecedent(s) but no child).

Variations in the density index according to this categorization reveal robust patterns: steady fields have a density of up to twice the average value, whereas ephemeral fields always have a below-average density. Emerging and declining fields feature intermediate values of mean density (*cf.* Fig. 5-b).

**Figure 6:**
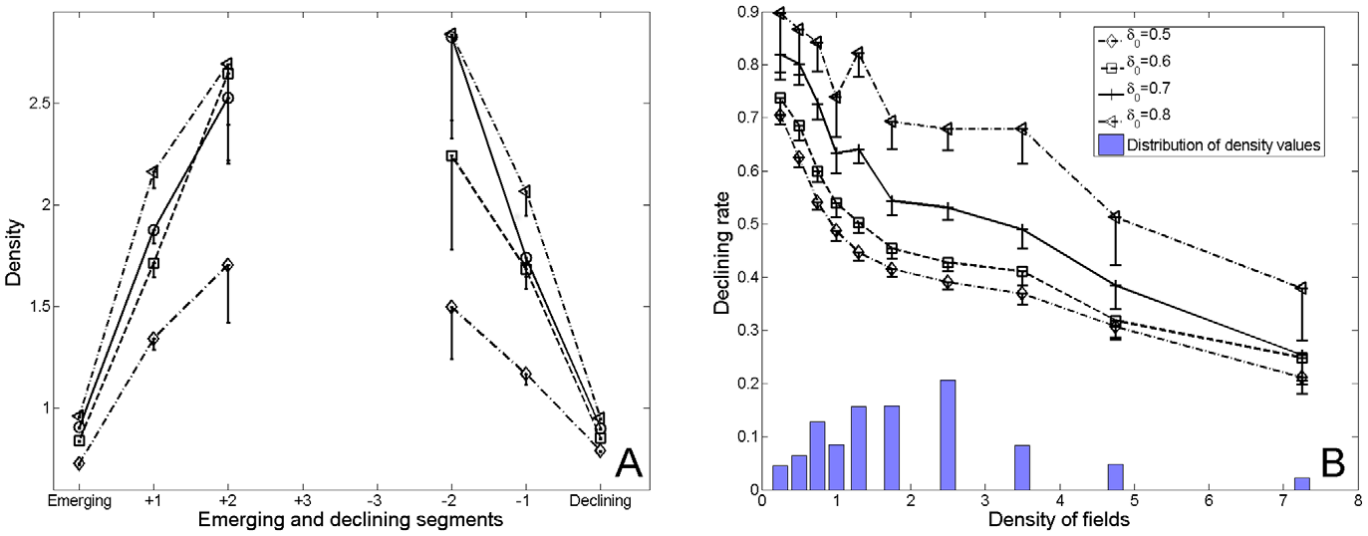
Relation between the density of fields and their sustainability. **A.** Variation of the mean density in the vicinity of emerging nodes and declining nodes. **B.** Empirical probability of a field being in decline, as a function of the density of the fields belonging to the phylomemetic network. Fields on emerging segments have been excluded from this analysis due to their specific density dynamics. The histogram represents the proportion of fields in each bin of densitity values. Error bars represent the 95% confidence interval.

This roof-shaped pattern suggests an initial trend in the life cycle of scientific fields: a growth in density when the field is emerging; a decrease when it starts to be neglected by the scientific community. This is confirmed by the density plot corresponding to emerging segments - starting with emerging nodes - and declining segments - ending with declining nodes - (see Fig. 6–a). Along a given sub-branch, starting at an emerging node, the mean density increases until the next special event. Conversely, the mean density decreases from the last special event until the declining node of the segment. These results support the idea of a gradient of sustainability, depending on the density of the fields. Mainstream science tends to take place at high-density values, whereas low-density research domains are more likely to disappear. This is well illustrated by Fig. 6–b, which plots the probability of a field to be declining, as a function of its density. Low-density fields have as much as 40% greater probability of declining than high-density fields, which raises the question of the predictability of science evolution.

**Figure 7:**
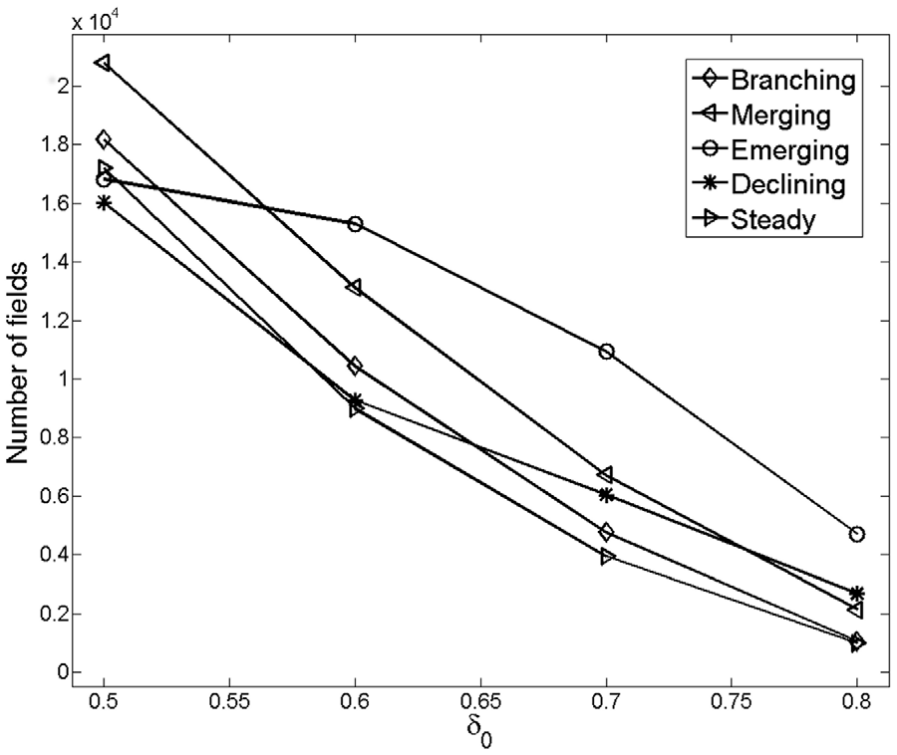
Variations of the number nodes in each category in function of 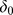. The phylomemetic network undergoes drastic changes in its composition for the studied range of 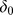 values, where the number of nodes in each category varies up to a factor 10.

It is noteworthy to mention that these patterns are very stable for 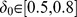, despite the fact that the phylomemetic network undergoes drastic changes in its composition for this range of values. Indeed, as shown on Fig. 7, the number of nodes in each category can vary up to a factor 10.

### The Jolting Routes of Paradigms

Whereas the evolutionary patterns appear to be quite simple along emerging or declining segments, Fig. 4 suggests that the evolution of scientific fields in-between these two extreme phases is far from linear: several branching and merging events punctuate their evolution, and undoubtedly represent important steps in the evolution of science.

**Figure 8:**
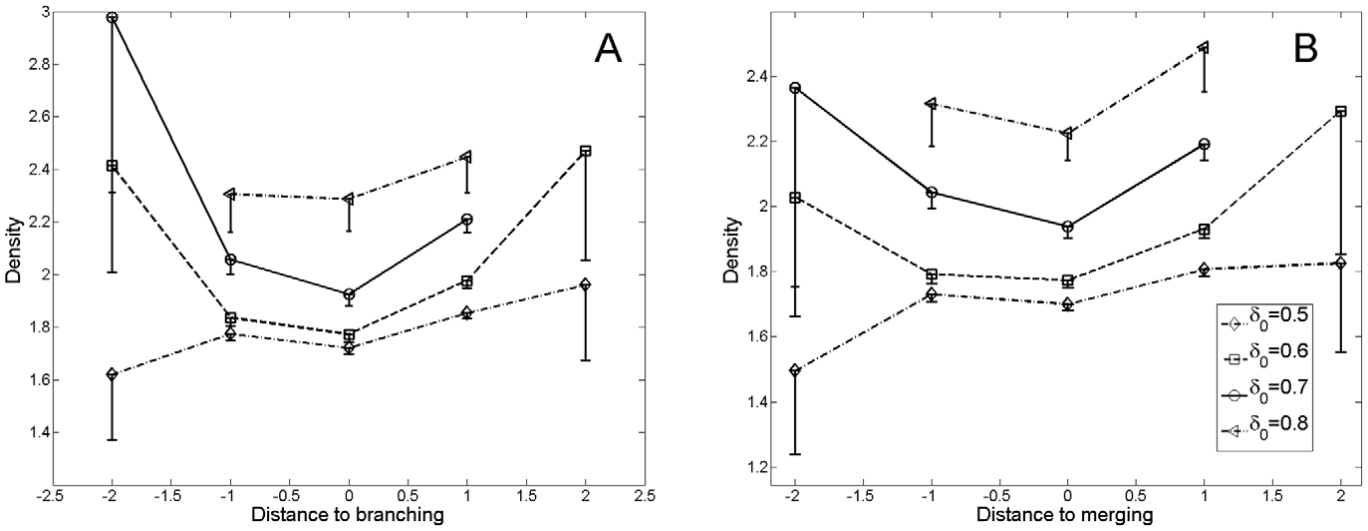
Evolution of the density in the vicinity of special events. **A.** Variation of density in the vicinity of a branching node. **B.** Variation of density in the vicinity of a merging node. Error bars represent the 95% confidence interval.

To gain a better understanding of what shapes the structure of the phylomemy and therefore the diversity of dynamic patterns encountered in science, we studied the variations in density around *special events* at the meso-level of clusters. After the removal of the branches covering less than 3 periods, which rendered the data noisy, we found a remarkable *U*-shaped curve indicating that, on average, the density reaches a local minimum at these critical points (*cf.* Fig. 8). The density significantly decreases before a special event takes place and increases during the following periods.

The underlying reason for the presence of a correlation between meso-structure variations and the density of scientific fields is undoubtedly multi-factorial. In particular, the fact that low-density values are not interpreted in the same manner for emerging segments as for other parts of the phylomemy (*cf.* Figs. 6-a and 6-b) suggests that a variety of factors are at stake, which have not all been taken into account in our study. The size of the community of scholars is one example.

In the present study, we investigated the insight provided by the micro-dynamics of terms, which may account for the observed meso-level patterns surrounding special events. We categorize a term 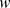 of a field 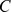, at a given period, according to the following possible classes:

- **Reconduction**: 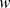 has already been used by some parents of 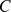,
- **Recombination**: 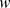 has previously been used in the phylomemy, but has not been used by any parent of 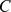,
- **Emergence**: 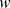 appears in the phylomemy for the first time.

By construction, the reconduction of some terms is clearly the rule in the phylomemy. This is the reason for which we prefer to focus on recombination and emergence processes, and compute the empirical probability of a scientific field being populated by such terms, as a function of its distance from branching and merging events. This quantity was then normalized on a yearly basis, to allow inter-temporal comparisons.

**Figure 9:**
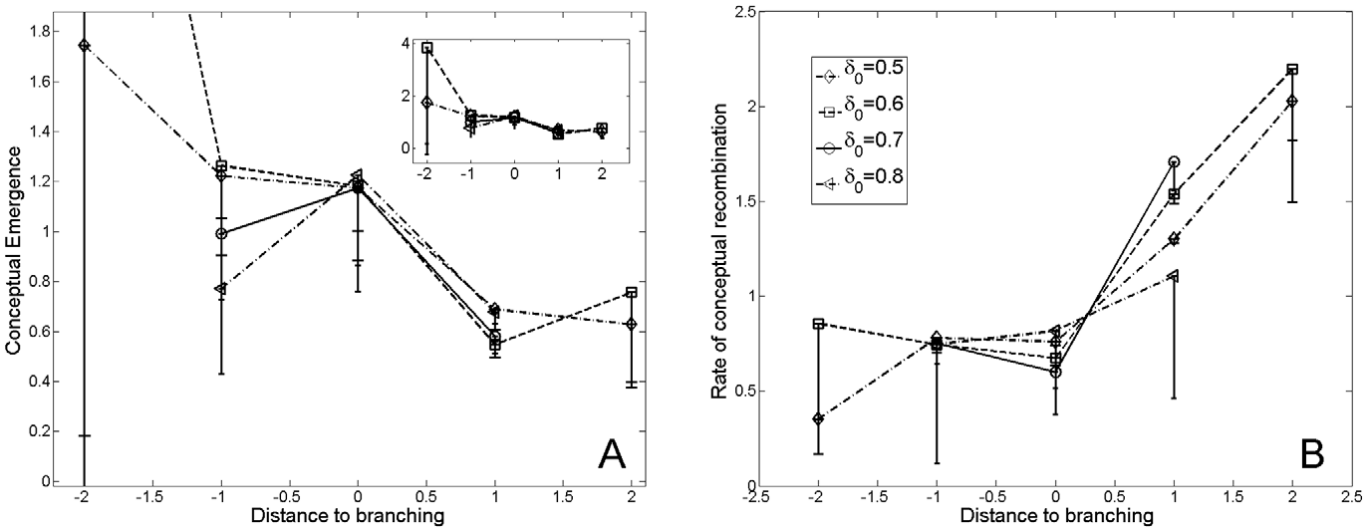
Variation of the rate of emergence (A) and conceptual recombinations (B) in the phylomemy, in the vicinity of a branching node. We observe a rate of conceptual emergence which is above average and quickly drops at the following period; whereas the proportion of recombinations is below average and quickly increases at the following period. Error bars represent the 95% confidence interval.

**Figure 10:**
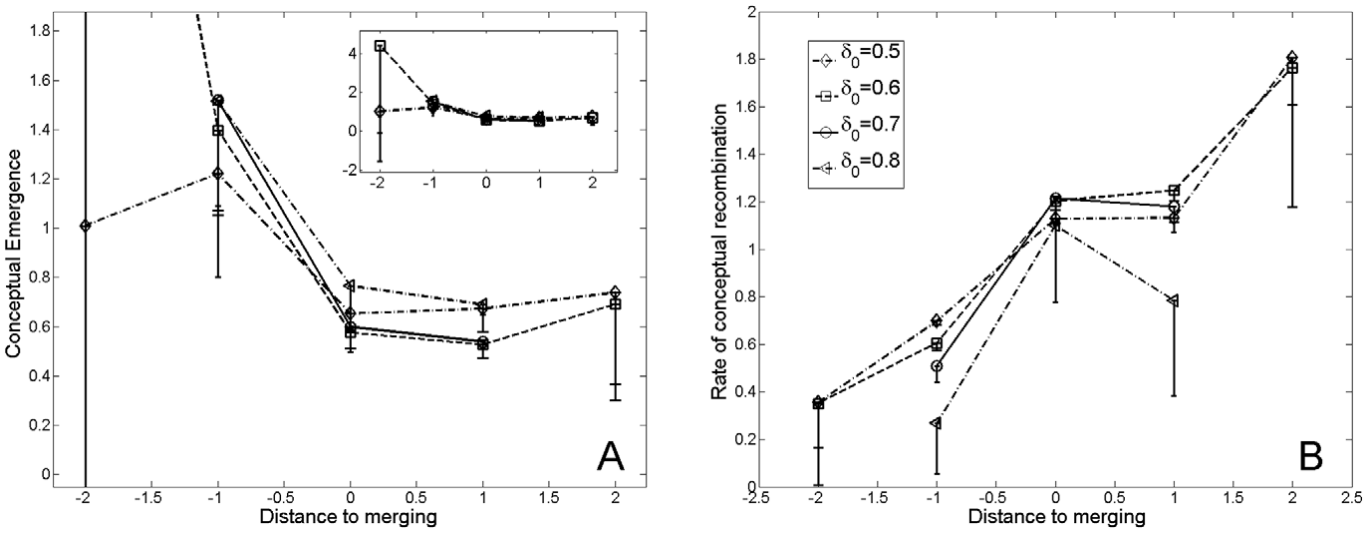
Variation of the rate of emergence (A) and conceptual recombinations (B) in the phylomemy, in the vicinity of a merging node. We observe a peak of conceptual emergence one period before merging, and a rate of conceptual emergence below average at merging nodes; while the rate of recombination is above average at merging nodes and is a monotonous increasing function in the vicinity of merging nodes. Error bars represent the 95% confidence interval.

The normalized rates of conceptual emergence and recombination in the phylomemy, in the vicinity of branching and merging events, are a bit noisy; nevertheless, they clearly differ suggesting differences in the terms-level dynamics for these two kind of events (*cf.* Fig. 9 and Fig. 10). At the branching nodes of the phylomemy, we observe a rate of conceptual emergence which is above average and quickly drops at the following period; whereas the proportion of recombinations is below average and quickly increases at the following period. On the contrary, for merging events, we observe a peak of conceptual emergence one period before merging, and a rate of conceptual emergence below average at merging nodes; while the rate of recombination is above average at merging nodes and is a monotonous increasing function in the vicinity of merging nodes.

## Discussion

This work extends the seminal work of Callon et al. (Callon et al., 1991) proposing a fully automatic method for the bottom-up reconstruction of the entire phylomemy of a scientific domain. We believe our approach can be useful both for the identification of scientific field transformations at a meso-level, and in the description of the dynamic patterns of science evolution. Indeed, we have shown that fields do not emerge, decay or hybridize at random: the likelihood of observing these dynamic events is significantly rooted in the structural properties of the fields observed at a given moment. The correlations we have computed show that, on average, scientific fields follow a prototypical lifecycle: long-living fields are being structured after their emergence, and may follow several branching or merging events, before decaying when their coherence decreases, which is measured here with the density index.

From our micro-dynamics analysis, we speculate that a loss of vitality in a given scientific field, which translates into a decrease in density, could be an incentive for scholars to change their strategies towards a more exploratory form of research. As observed in Fig. 9 and 10, scholars tends to focus on emerging terms before special events while the rate recombinations is below average. On the other hand, the rate of recombinations is above average right after special event, suggesting an effort to stabilize relations between scientific concepts. Thus, some special events could be interpreted as resilient steps in the evolution of scientific fields, in which a scientific community re-organizes its core concepts through the integration of emerging concepts.

Although further investigations would be required to appreciate the full range of evolutionary patterns and the various driving forces which define a field’s destiny, these preliminary results demonstrate the importance of special events, and the need to study several levels of organization - from the micro-level of terms to the global structure of the phylomemy - in order to propose comprehensive descriptions of the evolution of science.

We have shown that density can be a useful index for the description of a field’s viability. The level of emergences and recombinations in a field’s composition also appears to have significant importance. An investigation of the neighboring structure of fields would certainly provide new insight, which could be applied to the development of a general model allowing the dynamics of scientific fields to be predicted. In the present study, we chose to focus on scientific reconstruction in the perspective of a purely co-word analysis, whilst intentionally excluding many other dimensions such as: how are scientists populating these scientific fields?, is a given field still welcoming newcomers?, how are the citations generated in a given field structured?, and how do they relate to other fields?, etc. By taking these other dimensions into account, further light would undoubtedly be shed on science evolution. It is our hope that this first attempt will at least foster future efforts for the development of *quantitative epistemology* and the preparation of elaborate dynamic models based on the large-scale reconstruction of science phylomemies.

## Supporting Information

Supporting Information S1 **Details of the text-mining procedure.** Description of the complete processing of textual data. It relies on classical linguistic processes, at the end of which sets of candidate noun phrases are defined.
(PDF)

Supporting Information S2 **Comparison with results from a different dataset.** Presentation of a phylomemy reconstruction on a second dataset, performed in order to test the robustness of our results with respect to dataset variations and the quality of the terms list. This second data set, called “bio-networks”, is transdisciplinary. It comprises a biomedical corpus related to the research which recourses to the concept of networks. It includes approximately 140,000 articles extracted from Medline.
(PDF)

Supporting Information S3 **Study of the field size as an alternative coherence measure**. Study of the patterns related to the yearly normalized size of scientific fields for comparison to patterns in density for the embryo dataset.
(PDF)
