# Supplementary material for: Phylomemetic Patterns in Science Evolution—The Rise and Fall of Scientific Fields
Source: PLoS One. 2013 Feb 11;8(2):e54847. doi: 10.1371/journal.pone.0054847 (PMC3569444; doi:10.1371/journal.pone.0054847)
Supplement: Supporting Information S1 — Details of the text-mining procedure. Description of the complete processing of textual data. It relies on classical linguistic processes, at the end of which sets of candidate noun phrases are defined. (PDF) [file pone.0054847.s001.pdf]

SUPPORTING INFORMATION
Phylomemetic Patterns in Science Evolution
The Rise and Fall of Scientific Fields

David Chavalarias*†, Jean-Philippe Cointet*‡

SI.1 Details of the text-mining procedure

The complete processing of textual data can be described as follows. It first relies on classical linguistic processes, at the end of which sets of candidate noun phrases are defined:

1. **POS-tagging:** Part-of-Speech Tagging tool first tags every terms according to its grammatical type : noun, adjective, verb, adverb, etc. NLTK module was used extensively for this step.

2. **Chunking:** Tags are then used to identify noun phrases in the corpus. A noun phrase can be minimally defined as a pattern of successive nouns and adjectives. This step builds the set of our possible multi-terms.

3. **Normalizing:** We correct small spelling differences between multi-terms, arising from the presence/absence of hyphens. For example: we consider that the multi-terms "extra-cellular matrix", "extracellular matrix" and "extra cellular matrix" belong to the same class.

4. **Stemming:** Multi-terms can be combined if they share the same stem. Hence, singular and plural terms are automatically grouped into the same class (*e.g.* "carcinoma" and "carcinomas" are two possible forms of the stem: "carcinoma").

The grammatical constraints provide an exhaustive list of possible multi-terms grouped into stemmed classes, however we still need to select the N *most* relevant of these. Two assumptions are classically made in multi-word automatic term recognition tasks: relevant terms tend to appear more frequently, and longer phrases are more likely to be relevant. These criteria select the multi-terms which convey a certain semantic unit, that is to say those with the highest "unithood" (Van Eck et al., 2011). To sort the list of candidate terms we then apply a simple statistical criterium which entails the following steps:

- **Counting:** We enumerate every multi-term belonging to a given stemmed class in the whole corpus, in order to obtain their total number of occurrences (frequency). In this step, if two candidate multi-terms are nested, we increment the frequency of the larger chain only. For example, if "Insulin Growth Factor" is found in an abstract, we increment the multi-stem : "Insulin Growth Factor" only, but not the smaller stems such as "Growth Factor".

- **Unithood processing:** according to the method of Frantzi, K., & Ananiadou S. (2000) we associate each multi-stem with its *unithood*, defined as $u(i) = \log(l_i + 1)f_i$ where l_i is the number of terms involved in the multi-term *i* and f_i designates its frequency.

- **Pruning:** Items are then sorted according to their unithood, and the list is pruned to the $4 * N$ multi-stems with the highest C-value. This step removes less frequent multi-stems, but more importantly makes it possible to implement the following second-order analysis on the pruned list.

*Complex Systems Institute of Paris Ile-de-France (ISC-PIF), Paris, France
†CAMS, CNRS - EHESS, Paris, France
‡INRA-SenS, INRA, Marne-la-Vallée

- **Termhood processing:** Lastly, we adopt a similar approach to that of Van Eck et al. (2011), in order to remove irrelevant multi-terms with low termhood. Low termhood terms are neutral terms in a given corpus, that is to say which do not help characterizing the content of the text, although they may still occur very frequently, such as: “review of literature” or “past articles”. The rationale we follow is that irrelevant terms should have an unbiased distribution compared to other terms in the list, that is to say neutral terms may appear in any document in the corpus, whatever the precise theme they address. We first compute the co-occurrence matrix M between each item in the list, and then define the termhood of a multi-stem as the sum of the chi-square values it takes with all other classes in the list[1]. We rank the list according to its termhood and only the N most specific multi-stems are conserved.

For the embryo study, we chose $N = 2000$ without applying any further reduction to the data, whereas for the bio-net study, we chose $N = 1000$ and asked a science historian to eliminate irrelevant terms, which led to a final list of 834 terms.

References

[1] Frantzi, K., and Ananiadou, S. (2000) Automatic recognition of multi-word terms:. the C-value/NC-value method. International Journal on Digital Libraries

[2] van Eck, N. J., and Waltman, L. (2011) Text mining and visualization using VOSviewer. Arxiv preprint arXiv:1109.2058.

[1]More precisely, the termhood of a term i is defined as: $\theta(i) = \sum_{j \neq i} \frac{(M_{ij} - M_i M_j)^2}{M_i M_j}$ where $M_i = \sum_j M_{ij}$
