# Supplementary material for: Phylomemetic Patterns in Science Evolution—The Rise and Fall of Scientific Fields
Source: PLoS One. 2013 Feb 11;8(2):e54847. doi: 10.1371/journal.pone.0054847 (PMC3569444; doi:10.1371/journal.pone.0054847)
Supplement: Supporting Information S2 — Comparison with results from a different dataset. Presentation of a phylomemy reconstruction on a second dataset, performed in order to test the robustness of our results with respect to dataset variations and the quality of the terms list. This second data set, called “bio-networks”, is transdisciplinary. It comprises a biomedical corpus related to the research which recourses to the concept of networks. It includes approximately 140,000 articles extracted from Medline. (PDF) [file pone.0054847.s002.pdf]

SUPPORTING INFORMATION
Phylomemetic Patterns in Science Evolution
The Rise and Fall of Scientific Fields

David Chavalarias*†, Jean-Philippe Cointet*‡

SI.2 Comparison with results from a different dataset: use of the network concept in biology

This section presents a phylomemy reconstruction on a second dataset, performed in order to test the robustness of our results with respect to dataset variations and the quality of the terms list. For this dataset, the text-mining step is similar to text-mining step of the embryo dataset, but we choose to remove irrelevant terms.

This second dataset, called "bio-networks", is transdisciplinary. It comprises a biomedical corpus related to the research which recourses to the concept of networks. It includes approximately $140,000$ articles extracted from Medline[1] using the stem "netw" in their title or abstract. A list of 834 salient terms have been extracted from this corpus.

Terms list and co-occurrence data used for this dataset are available online[2].

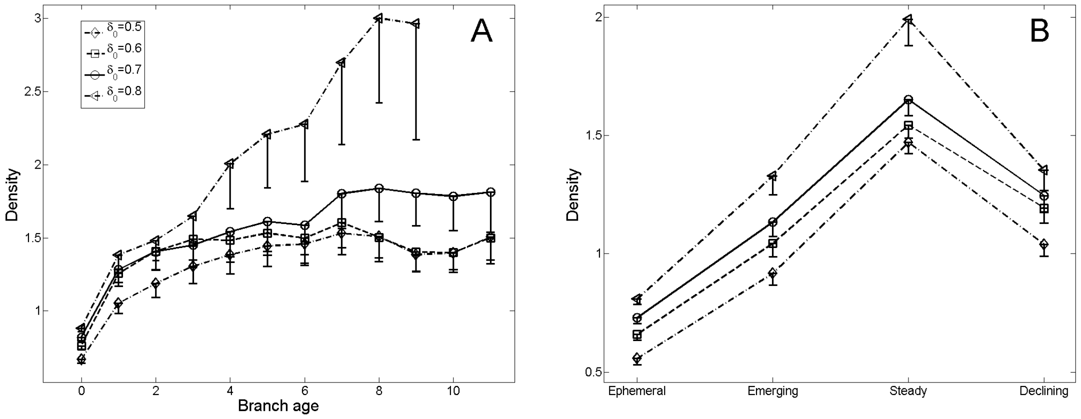

Fig. SI.2-1. Relation between fields density and their age for the "bionet" dataset. *A)* Variation of the mean density as a function of branch age, for different values of threshold δ_0. As for the "embryo" dataset, the density grows with branch age. *B* Dependence of the mean density on the fields position in the phylomemy. We observe the same roof-shaped pattern as for the "embryo" dataset. Error bars represent the 95% confidence interval.

*Complex Systems Institute of Paris Ile-de-France (ISC-PIF), Paris, France
†CAMS, CNRS - EHESS, Paris, France
‡INRA-SenS, INRA, Marne-la-Vallée

[1] http://www.ncbi.nlm.nih.gov

[2] http://hdl.handle.net/1902.1/19062

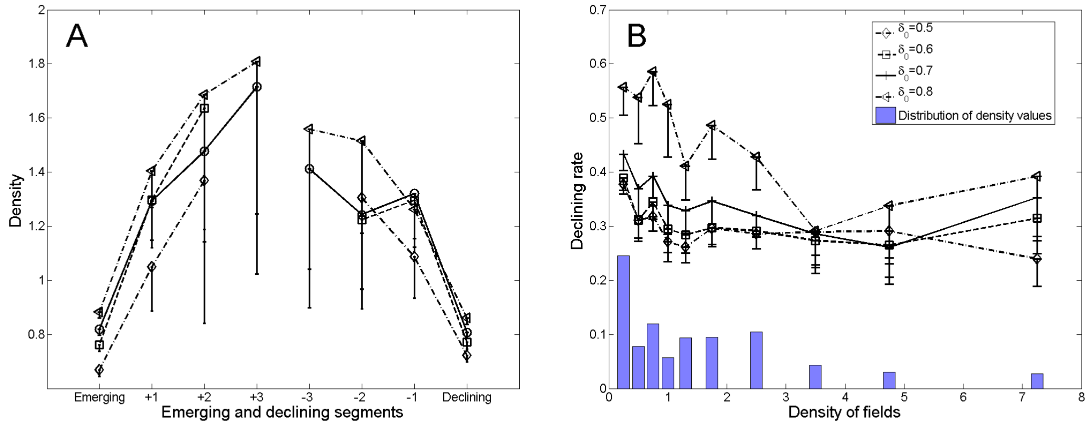

Fig. SI.2-2. Relation between the density of fields and their sustainability for the "bionet" dataset. *A)* Variation of the mean density close to emerging nodes and declining nodes. *B)* Empirical probability of a field being in decline, as a function of the density of the fields belonging to the phylomemetic network. Fields on emerging segments have been excluded from this analysis due to their specific density dynamics. Error bars represent the 95% confidence interval. We observe the same patterns as for the "embryo" dataset.

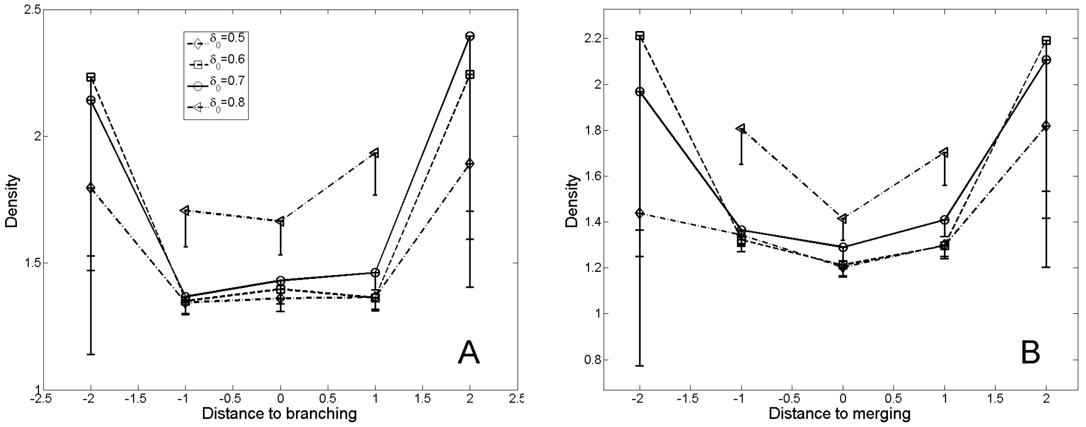

Fig. SI.2-3. Evolution of the density at the neighborhood of special events for the bionet dataset. A) Variation of density in the vicinity of a branching node. B) Variation of density in the vicinity of a merging node. Error bars represent the 95% confidence interval.

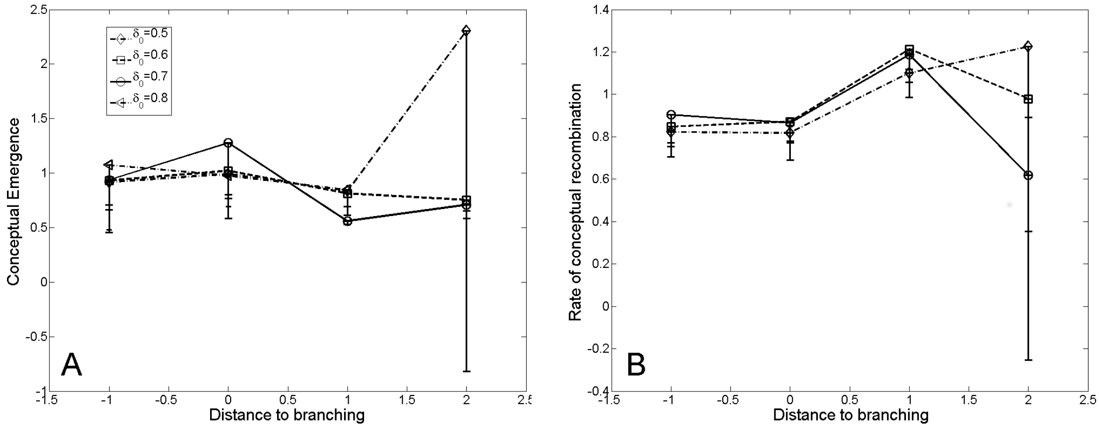

Fig. SI.2-4. Evolution of the rate of conceptual recombinations and emergences in the phylomemy at the neighborhood of a branching node. Variation of the rate of (A) emergences and (B) conceptual recombinations in the phylomemy, in the vicinity of a branching node. Error bars represent the 95% confidence interval.

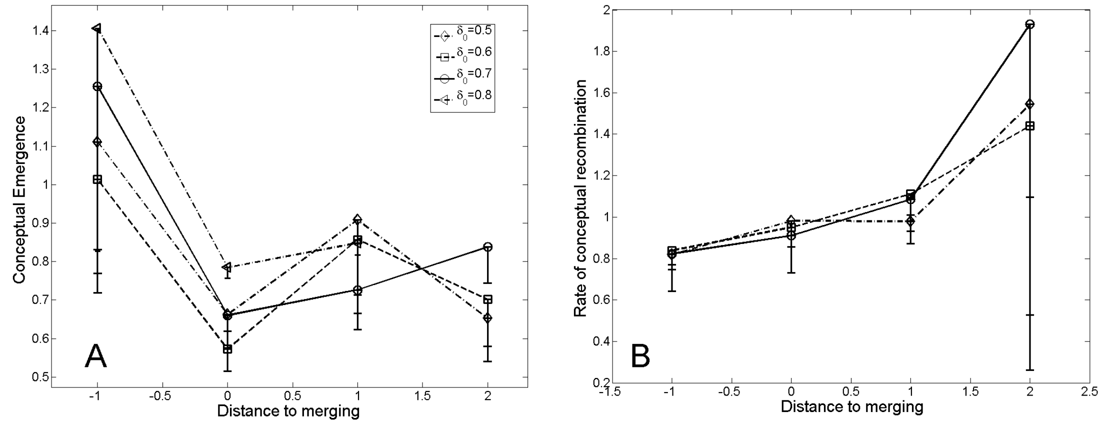

Fig. SI.2-5. Evolution of the rate of conceptual recombinations and emergences in the phylomemy at the neighborhood of a merging node. Variation of the rate of (A) emergences and (B) conceptual recombinations in the phylomemy, in the vicinity of a merging node. Error bars represent the 95% confidence interval.
