# Supplementary material for: Phylomemetic Patterns in Science Evolution—The Rise and Fall of Scientific Fields
Source: PLoS One. 2013 Feb 11;8(2):e54847. doi: 10.1371/journal.pone.0054847 (PMC3569444; doi:10.1371/journal.pone.0054847)
Supplement: Supporting Information S3 — Study of the field size as an alternative coherence measure. Study of the patterns related to the yearly normalized size of scientific fields for comparison to patterns in density for the embryo dataset. (PDF) [file pone.0054847.s003.pdf]

SUPPORTING INFORMATION
Phylomemetic Patterns in Science Evolution
The Rise and Fall of Scientific Fields

David Chavalarias*†, Jean-Philippe Cointet*‡

SI.3 Comparison with the field size as an alternative coherence measure

As a comparison with [1], we studied patterns related to the yearly normalized size of scientific fields (measured as the number of its terms) for the embryo dataset. Although fields size does exhibit clear patterns at an aggregated level, it seems less informative to understand finer-grained dynamics. For example, we observed that field size grows with branch age (*see* fig. SI.7), but is a poor predictor of the declining rate (*see* fig. SI.8)..

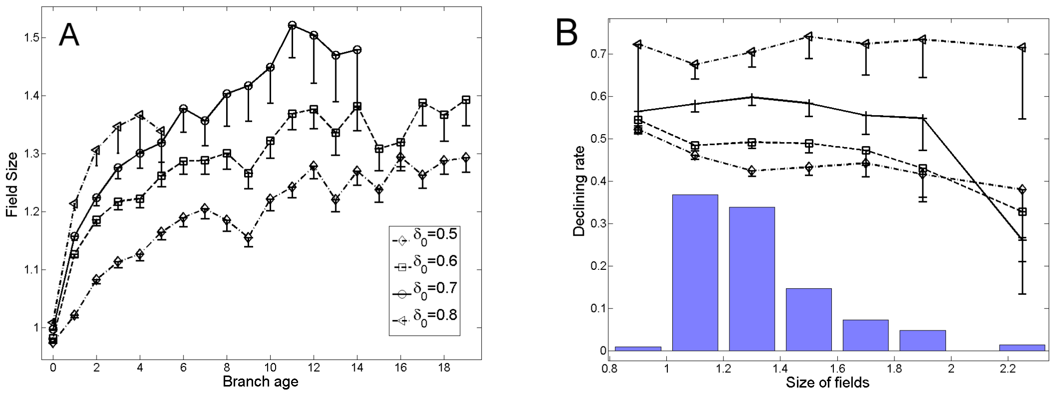

Figure SI.3-1. Some insight about the role of field size A) Variations of field size in the phylomemy depending on the branch age. Size has been normalized on a yearly basis. We observe that fields size clearly grows with branch age. B) Empirical probability of a field being in decline, as a function of its size. Fields on emerging segments have been excluded from this analysis for a better comparison with density patterns. Size has been normalized on a yearly basis. The histogram represents the proportion of fields in each bin of densitity values. We observe that there is little variation in declining rate with changes in fields size. Compared to density, fields size is thus a poorer indicator of their short term survival. Error bars represent the 95% confidence interval. Only lower bars are plotted for better visibility.

*Complex Systems Institute of Paris Ile-de-France (ISC-PIF), Paris, France
†CAMS, CNRS - EHESS, Paris, France
‡INRA-SenS, INRA, Marne-la-Vallée

References

[1] Herrera M, Roberts DC, Gulbahce N (2010) Mapping the evolution of scientific fields. PLoS ONE 5: e10355.
